# Assessing Vaccine Confidence Using the Vaccine Hesitancy Scale Among Adolescent Girls and Young Women at Risk of HIV Acquisition Living in Uganda, Zambia, and South Africa

**DOI:** 10.3390/vaccines13111083

**Published:** 2025-10-22

**Authors:** Nasimu Kyakuwa, Ali Ssetaala, Matt A. Price, Annet Nanvubya, Joel M. Abyesiza, Geofrey Basalirwa, Brenda Okech, Juliet Mpendo, Mubiana Inambao, Kawela Mumba-Mwangelwa, Chishiba Kabengele, Suzanna C. Francis, Pholo Maenetje, Ken Ondeng’e, Vinodh Edward, William Kilembe, Monica O. Kuteesa

**Affiliations:** 1 Uganda Virus Research Institute—International AIDS Vaccine Initiative (UVRI-IAVI) HIV Vaccine Program Ltd., Entebbe P.O. Box 49, Uganda; assetaala@iavi.or.ug (A.S.); ananvubya@iavi.or.ug (A.N.); gbasalirwa@iavi.or.ug (G.B.); bokech@iavi.or.ug (B.O.); jmpendo@iavi.or.ug (J.M.); 2 Department of Epidemiology and Biostatistics, University of California at San Francisco, San Francisco, CA 94122, USA; mattalexprice@gmail.com; 3 Medical Research Council/Uganda Virus Research Institute and London School of Hygiene and Tropical Medicine (MRC/UVRI & LSHTM) Uganda Research Unit on AIDS, Entebbe P.O. Box 49, Uganda; abyesizajoel@gmail.com; 4 Center for Family Health Research in Zambia (CFHRZ), Ndola 10101, Zambia; minambao@rzhrg-mail.org (M.I.); kmumba@rzhrg-mail.org (K.M.-M.); 5 Center for Family Health Research in Zambia (CFHRZ), Lusaka 10100, Zambia; ckabengele@rzhrg-mail.org (C.K.); wkilembe@rzhrg-mail.org (W.K.); 6 International AIDS Vaccine Initiative (IAVI), New York, NY 10004, USA; sfrancis@iavi.org (S.C.F.); kondenge@iavi.org (K.O.); 7 The Aurum Institute, Johannesburg 2001, South Africa; pmaenetje@auruminstitute.org (P.M.); vedward@auruminstitute.org (V.E.); 8 Department of Medicine, Vanderbilt University, Nashville, TN 37240, USA; 9 Uganda Virus Research Institute, Entebbe P.O. Box 49, Uganda; monakello@gmail.com

**Keywords:** vaccine confidence, vaccine hesitancy scale, vaccine hesitancy, vaccine uptake, adolescent girls, AGYW

## Abstract

Background: Vaccine hesitancy (VH) remains a major threat to global health that can reverse the progress in tackling vaccine-preventable diseases. Vaccine uptake among adolescent Girls and young women (AGYW) is often low. We assessed VH using a validated scale among AGYW in Uganda, Zambia, and South Africa. Methods: From June 2023 to February 2024, we recruited AGYW from fishing communities in Uganda, urban and peri-urban locations in Lusaka and Ndola, Zambia, and mining communities in Rustenburg, South Africa. Eligible participants were aged 15–24 years, sexually active, and HIV-negative but at risk for HIV acquisition. We collected demographic, HIV-related behavioral data, and vaccine hesitancy data using a structured questionnaire. Vaccine confidence was assessed using the 10-question Vaccine Hesitancy Scale that describes two factors, i.e., “vaccine confidence” and “risk tolerance”. Exploratory and Confirmatory Factor Analyses were performed to assess scale validity and internal consistency. Logistic regression was used to determine associations between demographics and vaccine confidence. Results: A total of 1213 AGYW participated in the study, with a mean age of 19.4 (SD ± 2.6) years. More than half (54%) were aged between 15 and 19 years. The majority of AGYW (94%) strongly believed that vaccines were important for their health and the community and that vaccination is a good way to protect them from diseases. About two-thirds of the AGYW (66%) indicated that they were concerned about the adverse effects of vaccines, while 30% responded that they did not need vaccines for diseases that were not common. We observed that 951 (78%) of the AGYW reported high vaccine confidence, while 494 (41%) reported low concerns over risks. Vaccine confidence varied across countries, with Zambia and Uganda showing lower vaccine confidence (adjusted odds ratios of 0.28 and 0.45, respectively, *p* < 0.005) in comparison to South Africa. Conclusions: A high level of vaccine confidence was observed among AGYW. Vaccine confidence among AGYW was driven more by the trust in vaccine safety and the need to protect communities against diseases. These findings suggest the potential for acceptance of vaccines, including future HIV vaccines, among AGYW. Despite high levels of vaccine confidence, concerns over vaccine risks remain substantial and must be addressed.

## 1. Introduction

Vaccine hesitancy (VH), defined as the delay in acceptance or refusal of vaccines despite the availability of vaccination services, remains a major threat to global immunization goals [1,2]. In East and Southern Africa, challenges in achieving high vaccination coverage rates persist, including VH, which undermines the effectiveness of vaccines critical for preventing high-burden diseases such as cervical cancer, hepatitis B, and COVID-19 [3,4,5]. Adolescent Girls and Young Women (AGYW) in this region face intersecting social, cultural, and informational vulnerabilities that shape their risk of VH [6]. These include limited autonomy in health decision-making, low levels of vaccine literacy, gender power dynamics that often prioritize community or parental and peer influence over individual agency [7,8], high exposure to misinformation, particularly through social media platforms [9,10,11], and low educational attainment and misconceptions about fertility and vaccine safety [12]. Additional influences include mistrust in health systems [13].

While VH is a global issue, its impact may be profound in vulnerable populations, particularly AGYW at risk of acquiring Human Immunodeficiency Virus (HIV). This is partly because they represent a crucial demographic at a high risk of vaccine-preventable diseases (VPDs), such as Human Papilloma Virus (HPV) and Hepatitis B and C [14]. VH may further hinder efforts to study, test, and deploy any future vaccines, including HIV vaccines.

The Strategic Advisory Group of Experts on Immunization (SAGE) acknowledges that many factors contribute to VH and that there is no unique group of determinants behind VH in all settings. According to the “3Cs” model, VH is linked to confidence, convenience, and complacency [2]. Confidence is defined as trust in the effectiveness and safety of vaccines; the system that delivers them, including the reliability and competence of health services and health professionals; and the motivations of policymakers who guide recommended vaccines. Convenience is defined as the perceived level of access to vaccinations. It depends on physical availability, affordability, geographical accessibility, ability to understand information (language and health literacy), and appeal of immunization services (the quality of the service). Complacency is defined as the perceived risk of contracting the disease; when the perceived risk is low, vaccination may be thought of as an unnecessary preventive action.

The long-term global goal is to develop a safe and effective HIV vaccine that protects people from acquiring HIV. Given the high vulnerability of AGYW, it is important to develop safe and effective HIV prevention products that could target this population. However, vaccine acceptance and confidence remain crucial barriers to the success of any vaccination program. Vaccine uptake among adolescents and young adults has been reported to be low [14], and this might impair the ability to study, test, and roll out any potential new HIV/AIDS vaccines.

Understanding the drivers of VH among AGYW in East and Southern Africa is essential to inform targeted, gender-responsive interventions to improve vaccine coverage. To understand VH among AGYW, the International AIDS Vaccine Initiative (IAVI) included a VH module in the Multisite study for AGYW for future HIV vaccines and antibodies for prevention of HIV (MAGY) study, conducted in Uganda, Zambia, and South Africa. The MAGY study partly aimed to establish cohorts of AGYW for the evaluation of HIV prevention products in sub-Saharan Africa. This publication presents findings from MAGY that focused on assessing vaccine confidence among AGYW.

## 2. Materials and Methods

### 2.1. Study Design

This cross-sectional survey was embedded within the MAGY study, a prospective observational cohort study. MAGY was a flagship study under the International AIDS Vaccine Initiative, Accelerate the Development of Vaccines and New Technologies to Combat the AIDS Epidemic (IAVI ADVANCE) program, enrolling AGYW (15–24 years old) between June 2023 and February 2024. Vaccine confidence data were collected from each participant as part of the baseline assessment at enrollment.

### 2.2. Study Setting

Participants were recruited from distinct communities in three countries: fishing communities around Lake Victoria (including islands Kimi and Nsazi, as well as landing sites Kasenyi, Kigungu, and Nakiwogo) in Uganda; urban and peri-urban areas in Lusaka and Ndola, Zambia, including primary healthcare settings serving single, sexually active mothers and known hotspots for female sex workers (FSWs); and various healthcare facilities, youth groups, and community outreach activities in Rustenburg, a mining town in South Africa’s North West Province. Before screening visits, community engagement was conducted to identify potential participants. Potential participants were invited to study clinics where screening and enrollment occurred. Additional recruitment strategies included peer referrals, participant recommendations, flyers and posters, and social media platforms such as Facebook and Twitter.

### 2.3. Study Participants

Eligible participants were HIV-negative, non-pregnant AGYW aged 15 to 24 years old, who reported sexual activity in the past three months, and met at least one criterion from a validated risk assessment questionnaire that adapted the VOICE risk assessment questionnaire [developed for adult women for Pre-exposure Prophylaxis (PrEP) trials in sub-Saharan Africa] [15], and the Ayton risk assessment (designed for AGYW in rural South Africa) [16]. HIV risk assessment was based on any one of the following: sexual intercourse in the past three months; use of contraception in the last year; perceived high HIV risk; ever having been pregnant; low HIV knowledge; financial dependence (relying on sexual partners for financial support); and any alcohol or illicit drug use in the past year. All participants who met the eligibility criteria were enrolled after providing informed consent.

### 2.4. Data Collection

Data collection took place in clinic rooms to ensure privacy and confidentiality. Trained study clinicians administered a face-to-face structured interview questionnaire (Supplementary File S1) to obtain social demographic data such as age, level of education, marital status, religion, source of income, and information about vaccines. Information about VH was obtained through administering the validated Vaccine Hesitancy Scale (VHS) [17], which included 10 Likert scale questions assessing thoughts on general vaccine confidence; responses were coded 1 for “strongly disagree”, 2 “disagree”, 3 “neither disagree nor agree”, 4 “agree”, or 5 “strongly agree”. The ten questions included; (1) Vaccines are important for my health; (2) Vaccines are effective; (3) Being vaccinated is important for the health of others in the community; (4) All routine vaccinations recommended by the local authority on vaccination (this varied by country) are beneficial; (5) New vaccines carry more risks than others; (6) The information I receive from the local authority on vaccination is reliable and trustworthy; (7) Getting vaccines is a good way to protect me from diseases; (8) Generally, I do what my doctor or healthcare provider recommends about vaccines for me; (9) I am concerned about serious adverse effects of vaccines; and (10) I don’t need vaccines for diseases that are not common anymore.

### 2.5. Statistical Analysis

The data were electronically captured in the REDCap (Westlake, TX, USA) software database, and data analysis was performed using STATA SE version 18 (Stata Corp, College Station, TX, USA). Participant characteristics were summarized overall and by study site.

To determine the latent traits or factors in the VHS, Exploratory Factor Analysis (EFA) was conducted on half of the sample (n = 606; randomly selected) using the Principal Component Factor method (PCF) and the maximum likelihood (ML) method for the factor loadings of the VHS with oblique rotation (Promax). Oblique rotation was chosen because the factors were expected to be correlated, allowing for a more accurate representation of the underlying structure. To examine model fit, Confirmatory Factor Analysis (CFA) was performed on the second half sample (n = 607). To determine the internal consistency, we used Cronbach’s alpha to determine scale reliability.

To determine the level of vaccine confidence for each item on the VHS, we constructed a 5-point scale of the class intervals for interpreting the VHS items’ average score. We reverse-coded items 1, 2, 3, 4, 6, 7, and 8 on the VHS to ensure that higher values consistently represent lower vaccine confidence. Scores (1–5) were grouped into class intervals to simplify analysis and interpretation. The interval width was calculated by dividing the score range (5 − 1 = 4) by the number of scores (5), resulting in a width of 0.8. Intervals were created by adding this width to the minimum score (1) (Table 1). Average scores, frequencies, and percentages were then calculated. This approach follows best practices by categorizing scores to improve interpretive clarity and facilitating comparisons [18].

A composite score for each respective factor was calculated by taking the mean values of its respective component questions. These scores were then dichotomized: values less than or equal to 2 (representing “Strongly Agree” or “Agree” responses, with regard to confidence in vaccines or risk tolerance) were coded as 0, while values greater than 2 (representing “Neither Agree nor Disagree,” “Disagree,” or “Strongly Disagree” responses) were coded as 1.

Bivariate logistic regression analyses were performed between covariates and both hesitancy scores (confidence and risk tolerance). We analyzed individual associations between demographic characteristics (including country, age, relationship status, religious affiliation, education level, source of income, and school attendance) and each outcome and calculated crude odds ratios with 95% confidence intervals and *p*-values. Covariates that showed statistical significance (*p* < 0.2) were then included in multivariate logistic regression models to identify factors independently associated with vaccine confidence. To control potential confounding factors, adjusted odds ratios were calculated for significant predictors.

## 3. Results

### 3.1. Socio-Demographic Characteristics of Study Participants

A total of 1213 AGYW were interviewed, 656 (54%) were aged between 15 and 19 years. The mean age was 19.4 (SD ± 2.6) years. The majority of AGYW, 1197 (99%), previously attended school, while only 351 (29%) were still in school. Most, 1107 (91%) of the AGYW had never married, and 750 (62%) were single with steady sexual partners. Details of the demographic characteristics are depicted in Table 2.

### 3.2. Responses to Vaccine Hesitancy Scale Items

The MAGY cohort showed strong positive beliefs about vaccines, with favorable mean scores regarding vaccines’ importance for personal health (1.77) and community benefit (1.78). They strongly agreed that vaccination is effective for disease prevention (1.72). However, they expressed significant concerns about vaccine safety, with a high mean score of 3.56 regarding serious adverse effects. They also showed moderate confidence towards new vaccines, perceiving them as riskier than established vaccines (mean score 2.74).

Most AGYW agreed or strongly agreed that vaccines were important for their health (94%); vaccines were effective (87%); being vaccinated was important for the health of others in the community (93%); and all routine vaccinations recommended by national vaccination programs were beneficial (91%). About two-thirds (66%) of the AGYW agreed or strongly agreed that they were concerned about the serious adverse effects of vaccines, while 30% agreed or strongly agreed that they do not need vaccines for diseases that are not common anymore. Details of the responses and average scores to the VHS items are shown in Table 3 below.

### 3.3. Structure, Model Fit, and Internal Consistency of the VHS

To examine the structure of our VHS items, we performed Exploratory Factor Analysis (EFA). Promax rotation was used in the EFA because it allows factors to correlate with each other, which is more realistic for behavioral constructs and helps identify a clearer factor structure. The Confirmatory Factor Analysis (CFA) remained unrotated since it tested a pre-specified factor structure based on theory, making rotation unnecessary. Details are shown in Table 4. The analysis revealed two distinct factors that describe the 10 VHS items, with Eigenvalues greater than 1. These two factors together accounted for 52% of the total variance in the items. We describe these two factors as “vaccine confidence” and “risk tolerance”. Vaccine confidence was dominant, explaining 40% of the variance, while risk tolerance explained 12%. Details are shown in Table 5. As shown in Table 4, 7 VHS items were loaded on vaccine confidence, and two items were loaded on risk tolerance. Only 9 of the 10 VHS items loaded on our factors. Item 9, “I am concerned about the serious adverse effects of vaccines,” didn’t load on either factor.

We conducted a CFA on two sets of the VHS items: one with nine items, excluding item 9 (“I am concerned about the serious adverse effects of vaccines”), and another with all ten items included. Using data from 607 participants, the analysis revealed that item 9 had a very weak loading of 0.14 on the risk tolerance factor. The CFA results demonstrated that all the remaining nine items loaded strongly onto their respective factors, providing robust support for our two-factor model, as detailed in the test statistics presented in Table 6.

To assess the internal consistency of both factors, we calculated Cronbach’s alpha based on data from 1213 participants. For vaccine confidence, Cronbach’s alpha was 0.85, indicating excellent scale reliability. However, for risk tolerance, Cronbach’s alpha was 0.44, which is considered poor. This low value is likely due to the small number of items on the risk tolerance factor, as scale reliability typically improves with more items. On including item 9 of our VHS on risk tolerance, our Cronbach’s alpha dropped to 0.34, implying that question 3 reduces the reliability of this factor. The correlation between the two factors was 0.26, suggesting a weak association and indicating that they represent separate dimensions of VH.

### 3.4. Relationship Between Demographic Characteristics and Vaccine Confidence

#### 3.4.1. Correlates of Vaccine Confidence

**Figure 1:**
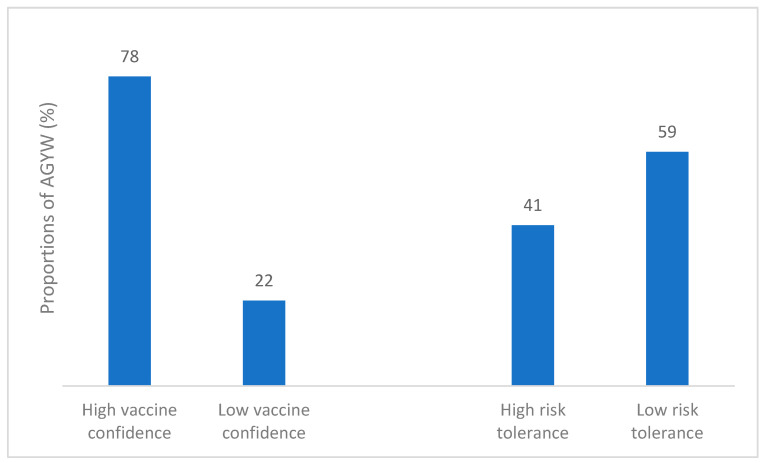
Vaccine confidence and Risk tolerance of 1213 AGYW.

As shown in Figure 1, 951 (78.4%) AGYW exhibited high vaccine confidence. We observed significant variations in vaccine confidence levels among countries, with AGYW in Zambia (adjusted odds ratios (aOR): 0.26, 95% CI: 0.18–0.39) showing a lower likelihood of vaccine confidence, followed by Uganda [aOR]: 0.44, 95% confidence interval (CI): 0.29–0.66) in comparison to South Africa. Participants not currently in school showed lower vaccine confidence compared to those who were in school (aOR 0.70, 95% CI: 0.50–0.97).

Participants with formal employment (aOR 0.55, 95% CI: 0.31–0.96) and those receiving Support/assistance (aOR 0.59, 95% CI: 0.40–0.87) showed lower vaccine confidence than the participants with no source of income. Table 7 shows the details of demographic characteristics and vaccine confidence.

#### 3.4.2. Correlates of Risk Tolerance

As shown in Figure 1, 41% of respondents demonstrated high risk tolerance. There was a significant variation in risk tolerance levels across the three countries, with Zambia (aOR: 0.22, 95% CI: 0.16–0.31) showing the lowest risk tolerance, followed by Uganda (aOR: 0.53, 95% CI: 0.37–0.76) compared to South Africa.

Participants in formal employment (aOR 0.44, 95% CI: 0.26–0.73), informal employment (aOR: 0.55, 95% CI: 0.33–0.94), and those receiving support/assistance (aOR 0.39, 95% CI: 0.26–0.60) showed significantly lower risk tolerance than the participants with no source of income.

Participants who were not in school showed lower risk tolerance compared to those who were in school (OR 0.66, 95% CI: 0.48–0.91). Details are shown in Table 8 below.

## 4. Discussion

Vaccination remains one of the most cost-effective strategies to reduce the global burden of infectious diseases. In this cohort of AGYW, we observed a high level of vaccine confidence coupled with a low risk tolerance for vaccines. Specifically, more than 90% of AGYW believed that vaccines were effective, safe, and that vaccination was important to protecting both themselves and their communities. These findings are promising from a public health perspective, as high vaccine confidence may lead to increased vaccine uptake and consequently reduce the burden of vaccine-preventable diseases. However, the low-risk tolerance noted could contribute to vaccine hesitancy, especially with the introduction of new vaccines.

A systematic review examining knowledge, attitudes, and practices towards adolescent vaccination in Africa reported high acceptability of vaccines among adolescents [19]. In contrast, Wang et al. reported lower levels of vaccine confidence among adolescents, who were less likely to believe in the benefits and safety of vaccines [20]. Notably, Wang et al.’s study included both adolescent males and females and found the males to be less confident about vaccines than the females. It also compared vaccine confidence among adolescents and adults, but never examined vaccine confidence among adolescents alone.

Despite ongoing vaccination promotion efforts, VH remains a significant issue [14,21] and was identified as one of the ten leading global health threats by the World Health Organization (WHO) [1]. To address this challenge, the WHO recommends regular monitoring of vaccine confidence, which remains understudied among adolescents [22,23]. Most of the recent studies have predominantly focused on vaccine confidence about COVID-19 vaccines [3,24], while others have targeted HPV vaccines [7,25,26,27,28]. Our study is among the first to assess vaccine confidence among AGYW at risk of HIV acquisition in sub-Saharan Africa using the VHS. The VHS has been widely used in different populations to assess VH and is more reliable in measuring “lack of confidence” than “risk tolerance” [29,30]. It demonstrated acceptable reliability and validity when applied to AGYW at risk of HIV, a finding similar to that of Shapiro et al. [17]. In our study, we found strong scale reliability for the “vaccine confidence” factor, with a high Cronbach’s alpha (0.85), while the “vaccine risk tolerance” factor showed poor reliability, with a Cronbach’s alpha of 0.44.

We observed several covariates correlating with vaccine confidence and tolerance for risk. AGYW living in Zambia were less likely to accept vaccines than those living in Uganda and South Africa. The AGYW from Zambia also demonstrated a lower risk tolerance for vaccines. This finding is not surprising, as vaccine confidence does vary by region and time [31,32]. Such geographical variation in vaccine confidence is expected, as it can be influenced by factors including healthcare accessibility, information availability, cultural beliefs, and trust in health systems [8,33]. Differences in vaccine information access are particularly relevant, as limited access may contribute to lower confidence [13,32].

While several studies have reported an association between the level of education and VH [34], we observed that there was no association between the level of education and vaccine confidence. For instance, Wegner et al. showed that Indian mothers with a high school education reported higher vaccine confidence than those with less [35]. The relationship between the level of education and VH is complex and influenced by various factors, including knowledge, perception, access to information, trust in healthcare systems, and sociocultural contexts [36]. AGYW with lower education levels may face challenges in accessing reliable health information or understanding and interpreting public health information. This could make them more susceptible to misinformation or confusion about vaccines, potentially contributing to VH. Furthermore, AGYW with no or less education might not fully understand the severity of vaccine-preventable diseases or may underestimate the potential risks of not vaccinating, leading to complacency [37]. This study, however, reported that AGYW who were not in school showed lower vaccine confidence compared to those who were in school. We did not observe any association between vaccine hesitancy (VH) and level of education, likely because more than three-fourths of the MAGY cohort were either currently in secondary school, had completed secondary school, or were enrolled in tertiary education. As a result, the educational status of participants was skewed toward AGYW with at least some high school education. Additionally, recruitment for the MAGY study began shortly after the peak of the COVID-19 pandemic, a period during which communities had experienced first-hand the life-saving impact of vaccines through the scale-up of COVID-19 vaccination efforts. This likely reduced the influence of formal education as the sole source of vaccine information, as community members were exposed to messaging on the benefits of vaccination from multiple sources beyond formal schooling. However, we did observe that AGYW who were currently in school had significantly higher vaccine confidence compared to those who were not. This may be attributed to the role that formal education played in vaccine education during the pandemic, reinforcing positive perceptions about vaccination.

We further report that the source of income was associated with vaccine confidence and risk tolerance. The AGYW who had no source of income were more likely to be vaccine-confident than those who were employed. AGYW with formal employment and those receiving support/assistance showed significantly lower vaccine confidence than the participants with informal or no source of income. The association between socioeconomic status and vaccine confidence is multifactorial [38]. Our finding that low socioeconomic status was associated with vaccine confidence could demonstrate the trust in healthcare systems, which in Africa often provide vaccines for free or at low cost, coupled with fewer resources to access alternative information sources that fuel VH (e.g., online misinformation) [39,40].

### Limitations

This study had some limitations. First, our study population was restricted to AGYW at risk for HIV, excluding AGYW who were pregnant, those living with HIV, and lower risk (typically those who did not report sexual activity in the previous three months), limiting generalizability. While the study benefits from a relatively large sample of diverse AGYW across three countries, our study population should not be considered broadly representative of Ugandan, Zambian, and South African AGYW. Second, “vaccine confidence” items on the VHS were worded positively, and all “risk tolerance” items were worded negatively. Consequently, the focus and content of the items on the scale became intertwined. Therefore, the item that was eliminated for not loading on either factor could have been due to the intertwining. Third, only two items loaded on the second factor assessing “tolerance for risks”. Scales with factors that are composed of fewer than three items are considered unstable, and calculating Cronbach’s alpha for a two-item sub-scale has limitations. Fourth, this study assessed responses to the VHS for vaccines in general; thus, these findings do not represent confidence in specific vaccines. It is well known that vaccine confidence varies according to the type of vaccine. Finally, this study was cross-sectional, and it is therefore not advisable to draw causal conclusions between our covariates and the respective correlated elements of confidence.

## 5. Conclusions

Our study reports that the VHS consisted of two factors, including “vaccine confidence” and “tolerance for risks.” However, the few items on risk tolerance could affect the scale’s reliability in measuring concerns and risks associated with vaccines. Vaccine confidence among AGYW was driven more by the trust in vaccine safety and the need to protect communities against diseases. This highlights the importance of addressing the perceptions and attitudes that the AGYW may have about vaccines, particularly newer ones. Demographic factors such as being in school, socioeconomic status, and country of origin were associated with vaccine confidence levels among AGYW in our study. Therefore, future interventions aimed at increasing vaccine uptake among AGYW should focus on improving education about vaccine safety tailored to the audience (e.g., cultural background, education, and socio-economic status), addressing specific concerns related to side effects, and leveraging trusted community leaders to build confidence in vaccines. Additionally, health communication strategies should be tailored to address the unique concerns of AGYW who may be more vulnerable to vaccine misinformation. This is crucial for informing future interventions aimed at enhancing vaccine uptake in this population.

## Figures and Tables

**Table 1:** The 5-point scale of the class intervals for interpreting the composite scores using averages (mean).

Class Interval/Interpretation (Level of Vaccine Confidence)	Interval
Very high vaccine confidence	1.00–1.80
High vaccine confidence	1.81–2.61
Moderate vaccine confidence	2.62–3.42
Low vaccine confidence	3.43–4.23
Very low vaccine confidence	4.24–5.04

**Table 2:** Socio-demographic characteristics of AGYW in the MAGY cohort study in Uganda, Zambia, and South Africa.

Demographic Characteristics	Uganda n (%)	Zambia n (%)	South Africa n (%)	Combined n (%)
Age (n = 1213) 15–19 20–24	217 (54.3) 183 (45.7)	234 (57.6) 172 (42.4)	205 (50.4) 202 (49.6)	656 (54.1) 557 (45.9) Mean 19.4 SD (2.6)
Relationship status (n = 1212) * Married Single with a steady partner Single with a casual partner(s) Single with no partners Others	33 (8.2) 223 (55.7) 117 (29.3) 1 (0.3) 26 (6.5)	7 (1.7) 209 (51.5) 174 (42.9) 3 (0.7) 13 (3.2)	1 (0.2) 318 (78.3) 78 (19.2) 8 (2.0) 1 (0.2)	41 (3.4) 750 (61.9) 369 (30.4) 12 (1.0) 40 (3.3)
Ever married (n = 1212) * Yes No	76 (19.0) 324 (81.0)	28 (6.9) 378 (93.1)	1(0.2) 405 (99.8)	105 (8.7) 1107 (91.3)
Religious affiliation (n = 1212) * Roman Catholic Protestant Born Again/Pentecostal Muslim/Islam Others	136 (34.0) 56 (14.0) 110 (27.5) 88 (22.0) 10 (2.5)	111 (27.3) 173 (42.6) 81 (20.0) 2 (0.5) 39 (9.6)	30 (7.4) 156 (38.4) 101 (24.9) 2 (0.5) 117 (28.8)	277 (22.9) 385 (31.8) 292 (24.1) 92 (7.6) 166 (13.7)
Currently in school (n = 1197 **) Yes No	61 (15.6) 329 (84.4)	116 (28.9) 285 (71.1)	174 (42.9) 232 (57.1)	351 (29.3) 846 (70.7)
Ever attended school (n = 1212 *) Yes No	390 (97.5) 10 (2.5)	401 (98.8) 5 (1.2)	406 (100) 0	1197 (98.8) 15 (1.2)
Parental status Yes No	200 (50.0) 200 (50.0)	101 (24.9) 305 (75.1)	120 (29.5) 287 (70.5)	421 (34.7) 792 (65.3)
Education level (n = 1197 **) Primary Secondary Tertiary/Higher education	159 (40.8) 216 (55.4) 15 (3.9)	107 (26.7) 284 (70.8) 10 (2.5)	3 (0.7) 362 (89.2) 41 (10.1)	269 (22.5) 862 (72.0) 66 (5.5)
Sources of income (n = 1212 *) None/no income Formal Employment Informal/alternative work Support/assistance	30 (7.5) 149 (37.3) 49 (12.3) 172 (43.0)	49 (12.1) 21 (5.2) 93 (22.9) 243 (59.9)	84 (20.7) 30 (7.4) 21 (5.2) 271 (66.8)	163 (13.5) 200 (16.5) 163 (13.5) 686 (56.6)

* Data for this variable were missing for 1 participant, ** Data for this variable were missing for 16 participants.

**Table 3:** Descriptive analysis of Vaccine Hesitancy Scale responses (n = 1213).

Likert Scale Items	Vaccine Hesitancy Scale Responses					Average Score and Interpretation
	SD (n%)	D (n%)	N (n%)	A (n%)	SA (n%)	
Vaccines are important for my health (R)	14 (1.2)	22 (1.8)	35 (2.9)	745 (61.4)	397 (32.7)	1.77 Very high vaccine confidence
Vaccines are effective (R)	15 (1.2)	69 (5.7)	73 (6.0)	722 (59.5)	334 (27.5)	1.94 High vaccine confidence
Being vaccinated is important for the health of others in the community (R)	12 (1.0)	29 (2.4)	39 (3.2)	732 (60.4)	401 (33.1)	1.78 Very high vaccine confidence
All routine vaccinations recommended the local authority on vaccination are beneficial (R)	5 (0.4)	41 (3.4)	61 (5.0)	761 (62.7)	345 (28.4)	1.85 High vaccine confidence
New vaccines carry more risks than others	92 (7.6)	511 (42.1)	279 (23.0)	280 (23.1)	51 (4.2)	2.74 Moderate vaccine confidence
The information I receive from the local authority on vaccination is reliable and trustworthy (R)	13 (1)	32 (3)	73 (6)	803 (66)	292 (24)	1.90 High vaccine confidence
Getting vaccines is a good way to protect me from disease (R)	9 (1)	11 (1)	32 (3)	739 (61)	422 (35)	1.72 Very high vaccine confidence
Generally, I do what my doctor or healthcare provider recommends about vaccines for me (R)	7 (1)	44 (4)	49 (4)	757 (62)	356 (29)	1.84 High vaccine confidence
I am concerned about the serious adverse effects of vaccines	28 (2)	261 (22)	120 (10)	618 (51)	186 (15)	3.56 Low vaccine confidence
I don’t need vaccines for diseases that are not common anymore	173 (14)	593 (49)	82 (7)	288 (24)	77 (6)	2.59 Moderate vaccine confidence

Key: SD: Strongly Disagree, D: Disagree, N: Neither Disagree nor Agree, A: Agree, SA: Strongly Agree, (R): Indicates items that were reverse-coded.

**Table 4:** Exploratory Factor Analysis, showing rotated and unrotated factor loadings (n = 606).

Vaccine Hesitancy Scale Items	Rotated EFA Loadings (Blanks for Values Less Than 0.32)		CFA Unrotated Loadings	
	Factor 1: Confidence	Factor 2: Risk Tolerance	Factor 1: Confidence	Factor 2: Risk Tolerance
Vaccines are important for my health (R)	0.68		0.75	
Vaccines are effective (R)	0.66		0.57	
Being vaccinated is important for the health of others in the community (R)	0.78		0.70	
All routine vaccinations recommended by the local authority on vaccination are beneficial (R)	0.68		0.71	
New vaccines carry more risks than others.		0.54		0.60
The information I receive from the local authority on vaccination is reliable and trustworthy (R)	0.53		0.68	
Getting vaccines is a good way to protect me from disease (R)	0.76		0.75	
Generally, I do what my doctor or healthcare provider recommends about vaccines for me (R)	0.67		0.54	
I am concerned about the serious adverse effects of vaccines.				
I don’t need vaccines for diseases that are not common anymore.		0.42		0.53

Note: EFA, Exploratory Factor Analysis. Method: maximum likelihood. Participants are randomly selected. 2 Factors. Rotation: oblique Promax (Kaiser off). CFA: Confirmatory Factor Analysis. Method: maximum likelihood.

**Table 5:** Exploratory Factor Analysis of putative latent factors (n = 606).

Factors	Eigen Value	Proportion
Factor 1	4.24	0.40
Factor 2	1.25	0.12
Factor 3	0.91	0.09
Factor 4	0.74	0.07
Factor 5	0.69	0.07
Factor 6	0.61	0.06
Factor 7	0.51	0.05
Factor 8	0.44	0.04
Factor 9	0.43	0.04
Factor 10	0.37	0.04

Note: Method: principal component factor method to describe latent factors in half the cohort (randomly selected). Retained 2 factors. We retain factors with eigenvalues greater than 1 (the Kaiser Criterion). No Rotation.

**Table 6:** Confirmatory Factor Analysis Model Fit statistics for a 2-factor model.

	Chi2	RMSEA	CFI	TLI	SRMR
Model 1 with 9 VHS items (Excluding item 9)	127.88	0.08	0.94	0.91	0.04
Model 2 with 10 VHS items	169.10	0.08	0.92	0.89	0.06
Value for good fit	Low value	<0.06	≥0.95	≥0.95	<0.08

Note. Chi2: Chi-Square Test Statistic, RMSEA: Root Mean Square Error of Approximation, CFI: Comparative Fit Index, TLI: Tucker–Lewis Index, SRMR: Standardized Root Mean Square Residual.

**Table 7:** Correlates of vaccine confidence.

Participant Demographic Characteristics	Bivariate Analysis			Multivariate Logistic Regression		
	OR	95% CI	*p* Value	aOR	95% CI	*p* Value
Country South Africa Uganda Zambia	Ref 0.37 0.26	0.26–0.51 0.18–0.37	<0.001 <0.001	Ref 0.44 0.26	0.29–0.66 0.18–0.39	<0.001 <0.001
Age 15–19 20–24	Ref 1.05	0.80–1.39	0.71			
Relationship status Married Single with a steady partner Single with a casual partner(s) Single with no partners Others	Ref 1.94 1.09 1.94 1.24	0.80–4.68 0.44–2.70 0.41–9.32 0.38–4.07	0.14 0.86 0.41 0.73			
Ever married Yes No	Ref 1.71	0.97–3.02	0.06	Ref 0.94	0.49–1.79	0.84
Religious affiliation Born Again/Pentecostal Protestant Roman Catholic Muslim/Islam Other	Ref 0.97 1.11 0.69 1.57	0.66–1.41 0.75–1.66 0.37–1.31 1.01–2.44	0.860 0.594 0.260 0.043			
Currently in school Yes No	Ref 0.53	0.40–0.71	<0.001	Ref 0.70	0.50–0.97	0.04
Ever attended school Yes No	Ref 0.56	0.12–2.48	0.44			
Parental status No Yes	Ref 0.69	0.51–0.92	0.01	Ref 0.79	0.56–1.11	0.18
Education level Primary Secondary Tertiary/Higher education	Ref 1.69 1.93	1.17–2.44 1.01–3.67	0.01 0.05	Ref 0.92 0.76	0.60–1.40 0.37–1.57	0.68 0.46
Sources of income None/no income Formal Employment Informal/alternative work Support/assistance	Ref 0.36 0.38 0.52	0.22–0.60 0.22–0.64 0.36–0.75	<0.001 <0.001 <0.001	Ref 0.55 0.69 0.59	0.31–0.96 0.39–1.22 0.40–0.87	0.03 0.21 0.01

OR: 95. CI: 95% confidence Interval.

**Table 8:** Correlates of risk tolerance.

Participant Demographic Characteristics	Bivariate Analysis			Multivariate Logistic Regression		
	OR	95% CI	*p* Value	aOR	95% CI	*p* Value
Country South Africa Uganda Zambia	Ref 0.48 0.22	0.36–0.66 0.16–0.29	<0.001 <0.001	Ref 0.53 0.22	0.37–0.76 0.16–0.31	<0.001 <0.001
Age 15–19 20–24	Ref 1.23	0.97–1.55	0.082	Ref 1.18	0.90–1.55	0.240
Relationship status Married Single with a steady partner Single with a casual partner(s) Single with no partners Others	Ref 1.18 0.79 2.12 0.96	0.62–2.23 0.41–1.51 0.50–9.03 0.40–2.32	0.620 0.469 0.307 0.925			
Ever married Yes No	Ref 0.89	0.59–1.34	0.561			
Religious affiliation Born Again/Pentecostal Protestant Roman Catholic Muslim/Islam Other	Ref 1.15 0.97 1.02 1.43	0.84–1.56 0.69–1.34 0.63–1.63 0.96–2.13	0.383 0.833 0.944 0.075			
Currently in school Yes No	Ref 0.66	0.51–0.86	0.002	Ref 0.66	0.48–0.91	0.011
Ever attended school Yes No	Ref 1.38	0.47–4.07	0.558			
Parental status No Yes	Ref 1.26	0.99–1.61	0.058	Ref 1.29	0.97–1.73	0.083
Education level Primary Secondary Tertiary/Higher education	Ref 1.41 2.28	1.07–1.85 1.27–4.09	0.015 0.006	Ref 0.98 1.07	0.71–1.34 0.56–2.06	0.885 0.836
Sources of income None/no income Formal Employment Informal/alternative Support/assistance	Ref 0.42 0.37 0.37	0.27–0.67 0.23–0.60 0.25–0.55	<0.001 <0.001 <0.001	Ref 0.44 0.55 0.39	0.26–0.73 0.33–0.94 0.26–0.60	0.002 0.028 <0.001

OR: Odds ratio, aOR: Adjusted Odds Ratio. 95% CI: 95% confidence Interval.

## Data Availability

The data presented in this study are available on request from the corresponding author. The data is not publicly available due to ethical restrictions.

## Supplementary Materials

The following supporting information can be downloaded at: https://www.mdpi.com/article/10.3390/vaccines13111083/s1, Supplementary File S1: Socio-demographic questionnaire. Vaccine Hesitancy module questionnaire.

## Abbreviations

The following abbreviations are used in this manuscript:

OR	Odds Ratio
95%CI	95% Confidence Interval
HIV	Human Immunodeficiency Virus
UVRI	Uganda Virus Research Institute
IAVI	International AIDS Vaccine Initiative
UNCST	Uganda National council for science and Technology
USAID	United States Agency for International Development
