# Supplementary material for: Assessing Vaccine Confidence Using the Vaccine Hesitancy Scale Among Adolescent Girls and Young Women at Risk of HIV Acquisition Living in Uganda, Zambia, and South Africa
Source: Vaccines (Basel). 2025 Oct 22;13(11):1083. doi: 10.3390/vaccines13111083 (PMC12656744; doi:10.3390/vaccines13111083)
Supplement: Supplementary file 1 [file vaccines-13-01083-s001.zip › vaccines-3865874-supplementary.pdf]

ALL RESPONSES ARE UNPROMPTED UNLESS SPECIFIED

Socio-demographic Module

In this first set of questions, we would like to ask you about a few things, including your education, where you live, and what you do for money.
Mukitundu kino ekisooka, twagala okukubuuza ebikukwataako ebitonotono okugeza obuyigirizeebwo, gy'obeera n'omulimu gw'okola.

**ALL RESPONSES ARE UNPROMPTED UNLESS SPECIFIED**		
SD01	Based on your screening, it looks like you are **<age in years calculated from screening CRF>** years old. Is this correct?<br><br>**Okusinziira kubikukwatako, kiraga nti olina emyaka <age in years calculated from screening CRF>. Kino kituuufu?**	1 Yes<br>2 No<br>99 Not Applicable (Paper CRF)
SD02	If no, please enter the correct date of birth here<br>**(Ask that they estimate if they don't know exactly. Fill out the month and day if you can)**	1 ____________________<br>DD/MMM/YYYY<br>99 Not Applicable (Paper CRF)
SD03	Where were you born?<br><br>**Wazaalibwa wa?**	1 ________________________Village / neighborhood / area<br>AND ___________Country<br>97 Don't know<br>96 Refuse to answer
SD04	Where do you currently live? **(Name of village / neighborhood / area)**<br><br>**Obeera wa mukiseera kino?**	1 ________________________ Village / neighborhood / area
SD05	How long have you been living continuously in the above-<br>mentioned village?<br><br>(Enter the number of years and/or months or tick the box if they've only just arrived) Enter 0 (zero) for years if less than one year.<br><br>**Omaze bbanga ki nga obeera mukitundu kino nga tovuddemu?**	1 __________ Years AND __________ Months<br>2 Just moved there in the last one month

ALL RESPONSES ARE UNPROMPTED UNLESS SPECIFIED		
SD06	What language (mother tongue) is<br>**best response)**<br>**Lulimi ki olunnansi olusinga**<br>most spoken at home? **(Choose**<br>**okwogerebwa ewaka wamwe?**	1 Luganda<br>2 Runyankole<br>3 Kinyarwanda<br>4 Kirundi<br>5 Lusoga<br>6 Rukiga<br>7 English<br>8 Kiswahili<br>98 Other, specify: ______________________________
SD07	What is your religious affiliation? **(Choose best response)**<br>**Oli wa ddiini ki?**	1 Roman Catholic<br>2 Protestant (e.g., Methodist, Anglican, etc.)<br>3 Born Again/Pentecostal<br>4 Moslem/Islam<br>5 Traditional / indigenous religion<br>6 No Religion, not religious<br>7 Other Christian, specify: ______________________<br>98 Other, specify: ______________________________

ALL RESPONSES ARE UNPROMPTED UNLESS SPECIFIED		
SD08	What is your tribe of birth?<br>**Oli wa ggwanga ki?**	1 Muganda<br>2 Munyankole<br>3 Mugishu<br>4 Musoga<br>5 Mukiga<br>6 Mutoro<br>7 Iteso<br>8 Munyoro<br>98 Other, please specify: __________
SD09	How many people live in your household, including children and adults?<br>**Bantu bameka ababeera mu maka gammwe ng'obaze abaana n'abantu abakulu?**	1 _____________ (Number of people)

ALL RESPONSES ARE UNPROMPTED UNLESS SPECIFIED		
SD13	If yes, how many financial dependents do you have? That is, people who are dependent on you for money, clothes, or food.<br><br>**Bwekiba yee, bali bameka b'olabirira mu ssente, eby'okwambala oba okulya?**	1 Enter number of financial dependents__________<br><br>99 Not Applicable
SD14	Who is the main woman (mother) in your home? Select the single best response:<br><br>**Mukyala ki omukulu mumaka gamwe?**	1 Self (no other main woman in the home)<br><br>2 Biological mother<br><br>3 Stepmother<br><br>4 Foster or adoptive mother<br><br>5 Grandmother<br><br>6 Aunt<br><br>7 Sister<br><br>8 Female cousin<br><br>9 Workmate<br><br>98 Other, specify: ____________________________
SD15	Does the main woman [in "main woman" question above] work or earn money?<br><br>**Omukyala omukulu akola oba alina omulimu oguyingiza ssente?**	1 Yes<br><br>2 No<br><br>97 Don't know/ not sure

ALL RESPONSES ARE UNPROMPTED UNLESS SPECIFIED		
SD16	Who is the main man in your home? Select the single best response:<br><br>**Musajja ki omukulu mumaka gamwe?**	1 No Male in home<br>2 Biological father<br>3 Stepfather<br>4 Foster or adoptive father<br>5 Sexual Partner<br>6 Grandfather<br>7 Uncle<br>8 Brother<br>9 Male cousin<br>10 Workmate
SD17	If there's a man in the home, does your main male caregiver [in "main man" above] work or earn money?<br><br>**Mumaka gammwe bwemuba mulimu omusajja 'omukulu' akola oba alina omulimu oguyingiza ssente?**	1 Yes<br>2 No<br>97 Don't know/ not sure<br>99 Not Applicable
SD18	Who usually makes decisions about the home/household?<br><br>**Ani atera okukola okusalawo mu maka gamwe?**	1 Myself<br>2 Spouse/boyfriend<br>3 Partner and I<br>4 Parent/Guardian/grandparents<br>98 Other, specify: ____________________

ALL RESPONSES ARE UNPROMPTED UNLESS SPECIFIED		
SD19	Which of the following best describes your home?<br><br>**Kubino wammanga kiriwa ekinnyonnyola obulungi amaka gamwe?**<br><br>**(Prompt the participant with each of the responses, select the best response)**	1 Mud hut<br><br>2 Brick / cinderblock house<br><br>3 Iron sheets house<br><br>4 Timber/ wooden house<br><br>98 Other Specify: ___________________
SD20	My house has running (piped) water on the property<br><br>**Amaka gaffe galina amazzi ga taapu**	1 Yes, inside the house<br><br>2 Yes, in the compound only<br><br>3 No
SD21	My house has private toilet facilities<br><br>**Amaka gaffe galina kabuyonjo eyaffe.**	1 Yes, inside the house<br><br>2 Yes, in the compound only<br><br>3 No
SD22	During the past 3 months **(Interviewer: describe back three months, or use a paper calendar to show the participant how long ago 3 months is)**, how many times have you spent a night or nights away from your residence in this community?<br><br>**Mu myezi esatu egiyise, mirundi emeka gy'omaze ekiro/ebiro wabweru w'amaka gamwe mu kitundu?**	1 Total number of times away_______________<br><br>2 Can't remember number of times away<br><br>99 Not Applicable
SD23	During the past 3 months **(Interviewer: describe back three months, or use a paper calendar to show the participant how long ago 3 months is)**, how many nights have you spent away from your residence in this community?<br><br>**Mu myezi esatu egiyise, biro bimeka by'osuze wabweru wamaka gamwe mu kitundu kino?**	1 Total number of nights away_______________<br><br>2 Can't remember number of times away<br><br>99 Not Applicable

ALL RESPONSES ARE UNPROMPTED UNLESS SPECIFIED		
SD24	What was the main purpose of spending a night/s away from your residence in this community? **(Select all that apply)**<br><br>**Nsonga ki eyakuviirako okumala ekiro/ebiro wabweru wamaka gamwe mukitundu kyamwe?**	1 Visiting friends or family<br>2 Visiting boyfriend/partner<br>3 School<br>4 Sex work (exchanging sex for money, goods, or services)<br>5 Other type of work<br>6 Trading<br>7 Seeking health care<br>98 Other, please specify: ____________________________
SD25	Have you ever attended school?<br><br>**Wasomako?**	1 Yes, I have attended some school<br>2 No, I have never attended school
SD26	What is your highest completed education level? (**Code highest level completed, i.e., only one answer allowed)**<br><br>**Wasoma kyenkana kyi?**	1 No formal education<br>2 Some Primary<br>3 Primary completed<br>4 Some Secondary<br>5 Secondary completed<br>6 Some or completed Technical Institute or vocational college<br>7 Some or completed University / college
SD27	Are you currently in school? (Or, if the student is on break, do they plan to resume school after the break?)<br><br>**Wetwogerera kati oli mussomero?**	1 Yes (including any current breaks)<br>2 No<br>99 Not Applicable

ALL RESPONSES ARE UNPROMPTED UNLESS SPECIFIED		
SD28	If yes, what type of schooling are you in? (**Prompt participant with the following choices, if school is currently on break, ask about their most recent term at school)**<br><br>**Bwekiba yee, kusoma kwa kika ki kw'olimu**	1 Boarding school (Attend school away from parents/guardians)<br>2 Regular day school (Attend school and come home daily)<br>3 Connecting with school remotely (i.e., internet-based schooling)<br>98 Other, specify: ___________________________<br>99 Not Applicable
SD29	How would you describe your employment or income situation? By this we mean, what do you do to get money (**Prompt participant with responses)**<br><br>**Oyinza kunnyonyola otya okukola kwo oba enfunayo? Kyetutegeeza, nti okola mulimuki okufuna ssente?**	1 I do not have a job at this time, and do not have a way to get money<br>2 I do not have a job at this time, but get money from parents/guardi ans<br>3 I do not have a job at this time, but get money from sexual partner(s)<br>4 Employed full time<br>5 I work regularly, but not full days (i.e., employed part time)<br>6 I do some work when I can (e.g., work during school breaks or holidays)
SD30	What has been your primary source of income in the last 12 months **(Interviewer: describe back three months, or use a paper calendar to show the participant how long ago 12 months is)**?<br><br>Please pick one from these options. **(Prompt participant with choices, note that some answers may be seen as sensitive…)**<br><br>**Wa w'obadde osinga okujja ssente mu bbanga ery'emyezi 12 egiyise?**	1 None / no income<br>2 Full time employment, 40 hours or more per week<br>3 Part time employment, less than 40 hours per week<br>4 Family/Relative (not sexual partner(s))<br>5 Spouse, boyfriend, sexual partners<br>6 Sex work (exchanging sex for money, goods, or services)<br>7 Social grants (South Africa Specific)<br>8 Bar work<br>9 Self-employment<br>10 Casual labor, describe: ______________________________<br>98 Other, specify:

ALL RESPONSES ARE UNPROMPTED UNLESS SPECIFIED		
SD31	Have you ever been married before?<br><br>**Wali ofumbiddwako?**	1 Yes, currently married<br><br>2 Yes, but now separated<br><br>3 Yes, but now divorced<br><br>4 Yes, but now widowed<br><br>5 No<br><br>96 Refuse to answer
SD32	How would you describe your current relationship status? **(Should be able to select more than one option, for example if participant is divorced, but has a new steady partner)**<br><br>**Oyinza kunnyonnyola otya bw'oyimiridde muby'omukwano /eby'obufumbo?**	1 Married (civil magistrate /traditional /religious)<br><br>2 Single but have a steady partner<br><br>3 Single but have casual partner(s)<br><br>4 Single with no partners now **(Interviewer probe: to enroll in the study they must be sexually active. Confirm what they mean by this response)**<br><br>98 Other, specify: ____________________________
SD33	**(If reports married, separated, widowed, or divorced)** At what age did you first get married?<br><br>**Walina emyaka emeka wewasookera okufumbirwa?**	1 _________________ Age in years<br><br>99 Not Applicable
SD34	**(If reports a partner)** Do you currently live with your sexual partner?<br><br>**Obeera n'omwagalwa wo mu kiseera kino?**	1 Yes<br><br>2 No<br><br>96 Refuse to say<br><br>99 Not Applicable (does not report a partner)
SD35	(If living with partner) how long have you lived with this partner?<br><br>**Omaze bbanga ki ng'obeera n'omwagalwawo?**	1 _____________________Time in months

	SIGNATURE	PRINTED NAME/INITIALS	DATE FORM SIGNED
Form Completed by:			
Reviewed by:			
First Entry by:			
Second Entry by:			

Vaccine Hesitancy module questionnaire

<table>
<tr><th colspan="3">Vaccine Hesitancy</th></tr>
<tr><td colspan="3">Read: Now we will ask you about vaccines in general, not just experimental vaccines for HIV. For each statement below, I would like you to tell me how much you agree with it. For each statement, your responses can be one of the following: Strongly Disagree, Disagree, Neither Agree nor Disagree, Agree, Strongly Agree

Kakati tugenda kukubuuza ku bikwata kukugema okwawamu, ssi okwokugezesa eddagala ery’okugema akawuka ka ssirimu. Ku buli ssitetimenti zino wammanga njagala onziremu ku ngeri gy’okkiriziganya nazo. Ojja kulonda
ku bino wammanga: Ssikiririza ddala, Ssikiriza, Tewali ku byombi, Nzikiriza, Nzikiririza ddala.

Interviewer: you may allow one and only one of the 5 Likert scale choices to be selected (Strongly Disagree / Disagree / Neither Agree nor Disagree/ Agree/ Strongly Agree) for each statement</td></tr>
<tr><td>VH1</td><td>Vaccines are important for my health

Eddagala erigema lyamugaso eri obulamu bwange</td><td>1 Strongly Disagree Ssikiririza ddala

2 Disagree Ssikkiriza

3 Neither Disagree nor Agree Tewali ku byombi

4 Agree Nzikiriza

5 Strongly Agree Nzikiririza ddala</td></tr>
<tr><td>VH2</td><td>Vaccines are effective</td><td>1 Strongly Disagree Ssikiririza ddala</td></tr>
</table>

Eddagala erigema likola

2	Disagree **Ssikkiriza**		
3	Neither Disagree nor Agree **Tewali ku byombi**		
4	Agree **Nzikiriza**		
5	Strongly Agree **Nzikiririza ddala**		

VH3	Being vaccinated is important for the health of others in my community<br><br>**Okwegemesa kyamugaso eri abantu abalala mukitundu kyange**	1<br><br>2<br><br>3<br><br>4<br><br>*5*	Strongly Disagree **Ssikiririza ddala**<br><br>Disagree **Ssikkiriza**<br><br>Neither Disagree nor Agree **Tewali ku byombi**<br><br>Agree **Nzikiriza**<br><br>Strongly Agree **Nzikiririza ddala**
VH4	All routine vaccinations recommended by the **Uganda National Expanded Program on Immunization** are beneficial<br><br>**Okugema kwonna okulambikiddwa [ekitongole ky'eggwanga ekirambika eby'okugema] kwa mugaso**	1<br><br>2<br><br>3<br><br>4<br><br>*5*	Strongly Disagree **Ssikiririza ddala**<br><br>Disagree **Ssikkiriza**<br><br>Neither Disagree nor Agree **Tewali ku byombi**<br><br>Agree **Nzikiriza**<br><br>Strongly Agree **Nzikiririza ddala**
VH5	New vaccines carry more risks older vaccines<br><br>**Eddagala erigema eppya lirina obulabe okusinga erisangiddwawo**	1<br><br>2<br><br>3<br><br>4<br><br>*5*	Strongly Disagree **Ssikiririza ddala**<br><br>Disagree **Ssikkiriza**<br><br>Neither Disagree nor Agree **Tewali ku byombi**<br><br>Agree **Nzikiriza**<br><br>Strongly Agree **Nzikiririza ddala**

VH6	The information I receive about vaccines from the **Uganda National Expanded Program on Immunization** is reliable and trustworthy<br><br>**Obubaka bwenfuna ku ddagala erigema okuva mu [ekitongole ky'eggwanga ekirambika eby'okugema] bwesigika**	1 Strongly Disagree **Ssikiririza ddala**<br><br>2 Disagree **Ssikkiriza**<br><br>3 Neither Disagree nor Agree **Tewali ku byombi**<br><br>4 Agree **Nzikiriza**<br><br>*5* Strongly Agree **Nzikiririza ddala**
VH7	Getting vaccines is a good way to protect me from disease<br><br>**Okwegemesa y'enkola ennungi ey'okuntangira okuva eri endwadde**	1 Strongly Disagree **Ssikiririza ddala**<br><br>2 Disagree **Ssikkiriza**<br><br>3 Neither Disagree nor Agree **Tewali ku byombi**<br><br>4 Agree **Nzikiriza**<br><br>*5* Strongly Agree **Nzikiririza ddala**
VH8	Generally, I do what my doctor or healthcare provider recommends about vaccines for me<br><br>**Okutwaliza awamu, nkola ebyo omusawo wange byaba angambye ku bikwatagana kukwegemesa.**	1 Strongly Disagree **Ssikiririza ddala**<br><br>2 Disagree **Ssikkiriza**<br><br>3 Neither Disagree nor Agree

VH9	I am concerned about serious adverse effects of vaccines<br><br>**Ndi mweralikirivu kubulabe obuva mu ddagala erigema**	1 Strongly Disagree<br>**Ssikiririza ddala**<br><br>2 Disagree<br>**Ssikkiriza**<br><br>3 Neither Disagree nor Agree<br>**Tewali ku byombi**<br><br>4 Agree<br>**Nzikiriza**<br><br>*5* Strongly Agree<br>**Nzikiririza ddala**
VH10	I do not need vaccines for diseases that are not common anymore<br><br>**Ssetaaga kwegemesa ndwadde ezitakyali zabulijjo**	1 Strongly Disagree<br>**Ssikiririza ddala**<br><br>2 Disagree<br>**Ssikkiriza**<br><br>3 Neither Disagree nor Agree<br>**Tewali ku byombi**<br><br>4 Agree<br>**Nzikiriza**<br><br>*5* Strongly Agree<br>**Nzikiririza ddala**

	SIGNATURE	PRINTED NAME/INITIALS	DATE FORM SIGNED
Form Completed by:			
Reviewed by:			
First Entry by:			
Second Entry by:			
